# Interfacing with Neural Activity via Femtosecond Laser Stimulation of Drug-Encapsulating Liposomal Nanostructures

**DOI:** 10.1523/ENEURO.0107-16.2016

**Published:** 2016-11-16

**Authors:** Takashi Nakano, Sean M. Mackay, Eng Wui Tan, Keshav M. Dani, Jeff Wickens

**Affiliations:** 1 Neurobiology Research Unit, Okinawa Institute of Science and Technology Graduate University, Onna-son 904-0412, Okinawa, Japan; 2 Department of Neurobiology, Institute of Biomedical and Health Sciences, Hiroshima University, Hiroshima 734-8553, Japan; 3 Department of Chemistry, University of Otago, Dunedin 9016, New Zealand; 4 Femtosecond Spectroscopy Unit, Okinawa Institute of Science and Technology Graduate University, Onna-son 904-0495, Okinawa, Japan

**Keywords:** femtosecond, laser, liposome, nanoshell, neurotransmitter, release

## Abstract

External control over rapid and precise release of chemicals in the brain potentially provides a powerful interface with neural activity. Optical manipulation techniques, such as optogenetics and caged compounds, enable remote control of neural activity and behavior with fine spatiotemporal resolution. However, these methods are limited to chemicals that are naturally present in the brain or chemically suitable for caging. Here, we demonstrate the ability to interface with neural functioning via a wide range of neurochemicals released by stimulating loaded liposomal nanostructures with femtosecond lasers. Using a commercial two-photon microscope, we released inhibitory or excitatory neurochemicals to evoke subthreshold and suprathreshold changes in membrane potential in a live mouse brain slice. The responses were repeatable and could be controlled by adjusting laser stimulation characteristics. We also demonstrate the release of a wider range of chemicals—which previously were impossible to release by optogenetics or uncaging—including synthetic analogs of naturally occurring neurochemicals. In particular, we demonstrate the release of a synthetic receptor-specific agonist that exerts physiological effects on long-term synaptic plasticity. Further, we show that the loaded liposomal nanostructures remain functional for weeks in a live mouse. In conclusion, we demonstrate new techniques capable of interfacing with live neurons, and extendable to *in vivo* applications.

## Visual Abstract

**Figure:**
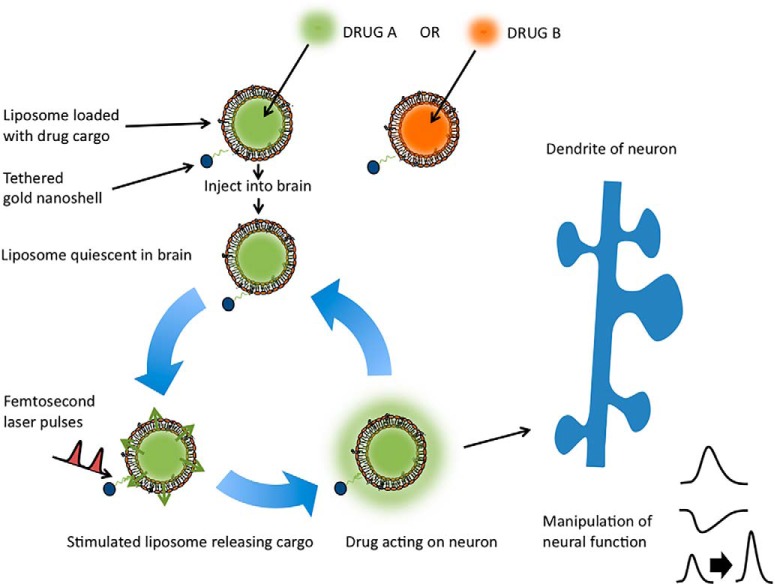

## Significance Statement

We describe a novel neural manipulation method using laser and liposomal nanostructures. The method enables us to release various types of neurochemicals and drugs in the mouse brain, beyond the range used by existing optical manipulation methods, such as optogenetics and caged compounds. We also demonstrate repeated and stable neural manipulation modulated by laser intensity. Given the established biocompatibility and stability of liposomes in the body, these findings suggest that the liposomal neural manipulation methods would be a useful tool for neuroscience research and further treatment of neurological disorders.

## Introduction

The development of optical methods for manipulating neurons has revolutionized the investigation of causal relationships between neural activity and function, with the potential to add to the understanding of mechanisms and the treatment of neurological disorders. A particular strength of optical methods is the ability to interact with neurons with fine spatiotemporal resolution (Callaway and Yuste, 2002; Szobota and Isacoff, 2010; Shepherd, 2012; Lim et al., 2013), but existing methods have some important limitations. Until now, caged compounds and optogenetic methods have been the principal methods of optical manipulation. Caged compounds are made by bonding caging moieties with target chemicals, such as calcium, glutamate (Glu), and dopamine (Ellis-Davies, 2007). When optically stimulated, the caged compounds undergo photolysis and release the target chemicals. Uncaging techniques, however, are limited in the range of compounds that can be caged and may require complex synthesis procedures. In addition, they are limited to “one-shot” nonrepeatable release from each caged molecule. The more recent optogenetic method allows optical manipulation of neurons that are genetically modified to express light-activated protein (Papagiakoumou et al., 2010; Yizhar et al., 2011). Optogenetic stimulation can both activate and inhibit neurons, and, thus, can control neurotransmitter release from neurons. However, optogenetic stimulation is limited to releasing neurotransmitters that are naturally synthesized by genetically targeted neurons. Due to these limitations, no existing method can stimulate the release of arbitrarily selected neurochemicals and drugs with amplitude controlled locally, repeatedly, and rapidly. We here report a new optical method enabling the use of a more complete pharmacopoeia of neurochemicals and drugs to specifically stimulate or inhibit particular receptors or ion channels, with high spatiotemporal resolution and repeatability.

In previous work, a new method of chemical release from liposomal nanostructures using optical stimulation was described (Wu et al., 2008; Leung and Romanowski, 2012; Nakano et al., 2014; Huang et al., 2015). Liposomal nanostructures are submicron spherical capsules comprising a lipid bilayer shell and an interior space that can be loaded with drugs. By tethering a gold nanoparticle to the liposome wall, the repeatable release of the encapsulated drug from the liposome can be effected by exposure to femtosecond laser pulses (Nakano et al., 2014). By adjusting laser stimulation parameters, the amount and speed of release can be controlled. In addition to the biocompatibility and capability to encapsulate many types of chemicals, this method has the favorable characteristic of providing rapid, on-demand, and repeatable delivery on a physiological timescale. Although these “in-beaker” results indicate the potential of this method for the manipulation of neural activity in neuroscience research, until now it has not been applied in living brain tissue.

In this study, we demonstrate experimental manipulation of neural activity in live mouse brain tissue by laser-stimulated release of neurochemicals from liposomal nanostructures, releasing a range of different neurochemicals, drugs, and metal ions. We also show that the amount of release from liposomes can be controlled, and that the release is repeatable and reliable. Importantly, we demonstrate the release of a wider range of chemicals than is possible using optogenetics or uncaging. In particular, we show that the release of a pharmacological agent, a synthetic receptor-specific agonist, could exert physiological effects on long-term synaptic plasticity. These experiments demonstrate new possibilities for neural manipulation based on liposomal nanostructures.

## Materials and Methods

### Slice preparation

Animals were handled in accordance with protocols approved by the animal care and use committee of authors' university. Experiments were performed on male Drd1a-eGFP BAC transgenic mice bred on a Swiss Webster background (inbred; The Jackson Laboratory) and a C57BL/6 cross [age, postnatal day 30 (P30) to P80]. Animals were deeply anesthetized with isoflurane and decapitated, and the brain was removed rapidly. Horizontal slices, 300 mm thick, containing the hippocampus and entorhinal cortex or oblique horizontal corticostriatal slices were cut on a vibratome (VT1200S, Leica Microsystems) in a cold cutting solution containing the following (in mm): 92.0 *N*-methyl-d-glucamine, 2.5 KCl, 10.0 MgCl2, 0.5 CaCl2, 1.25 NaH2PO4, 30.0 NaHCO3, 20.0 HEPES, 2.0 thiourea, 5.0 sodium ascorbate, 3.0 sodium pyruvate, and 25.0 glucose, and saturated with 95% O_2_/5% CO_2_ (VT1200S, Leica). Slices were then incubated in oxygenated artificial CSF (ACSF) maintained at a temperature of 36°C for 1 h. The standard ACSF had the following composition (mm): 118.0 NaCl, 2.5 KCl, 2.0 CaCl2, 1.0 MgCl2, 26.0 NaHCO3, 1.25 NaH2PO4, 1.5 myo-inositol, 0.5 sodium ascorbate, 2.0 sodium pyruvate, and 10.0 glucose. After incubation, a single slice was transferred to a recording chamber placed on the stage of an upright microscope, and perfused (3–4 ml/min) with oxygenated ACSF at 32°C. The remaining slices were kept in a holding chamber containing oxygenated ACSF at room temperature until required.

### Electrophysiological recording

Whole-cell recordings were made from CA1 pyramidal neurons in hippocampus or the D1-type dopamine receptors expressing medium spiny neurons in the striatum. Patch pipettes (4–6 MΩ) were filled with internal solution containing the following (in mm): 132.0 K gluconate, 6.0 KCL, 6 NaCl2, 10.0 HEPES, 2 MgCL2, 2.0 NaATP, 0.4 NaGTP, and 0.5 EGTA, pH 7.2–7.4. Signals were amplified by MultiClamp 700B (Molecular Devices), digitized at 10,000 Hz and filtered with a band of 1–2,000 Hz by pCLAMP 10 (Molecular Devices). Off-line analysis was conducted using MATLAB (MathWorks).

### Preparation of liposomal nanostructures

Drugs and chemicals that were encapsulated in liposomes were all dissolved in Dulbecco’s PBS (catalog #14040133, ThermoFisher Scientific) as follows: Glu, 100 mm l-glutamic acid (Tocris Bioscience); carboxy-fluorescein (CF), 1 mm 6-carboxy-fluorescein (Bachem Feinchemikalien AG); potassium chloride (KCl), 2.6 M KCl plus 1 mm CF; muscimol, 100 mm muscimol (Tocris Bioscience) plus 1 mm CF; D1 Agonist (SKF), 12 mm SKF-38393 hydrochloride (Tocris Bioscience) plus 36 mm l-ascorbic acid sodium salt (Nacalai Tesque); and ascorbic acid, 36 mm l-ascorbic acid sodium salt (Nacalai Tesque).

Liposomal nanostructures were assembled from liposomes, tethering molecules, and hollow gold nanoshells (HGNs). To prepare the liposomes, the following compounds were combined in a flask in the molar ratios of 100:5:5:4:3.5, 1,2-distearoyl-sn-glycero-3-phosphocholine (Echelon Biosciences), cholesterol (Nacalai Tesque), sphingomyelin (Avanti Polar Lipids), 1,2-distearoyl-sn-glycero-3-phosphoethanolamine-N-[methoxy(polyethylene glycol)-2000] (DSPE-PEG2000, Avanti Polar Lipids), and the tethering molecule DSPE-PEG2000-SH dissolved in chloroform (Nacalai Tesque). A thin film of this lipid mixture was then deposited on the surface of the flask by heating and evaporating the lipid mixture under reduced pressure while warming the flask in warm water. The thin lipid film was suspended in a buffer solution (0.8 ml PBS) containing the drug to be encapsulated by swirling in a water bath at 50°C until all the lipid material was suspended. Immediately afterward, uniformly sized liposomes were obtained by extrusion of the suspension through a 400 nm polycarbonate membrane at a temperature above the transition temperature of the lipids. Based on a study of filter-extruded liposomes by Hinna et al. (2016), we expect that most of the liposomes are unilamellar with a minority of bilamellar (multiwalled) ones in the mix. The suspension of tether-ready liposomes was then placed in a sample vial (15–20 ml capacity).

To link the gold nanoshells, the HGNs were first suspended in HEPES buffer, 10 mm, pH 7.3, gold 8.1 mg/ml. The diameter of the HGNs was ∼20 nm with absorption in the visible and near-infrared regions. The HGN suspension was added to the liposome suspension in aliquots of 2.9 µl while the solution was agitated on a vortex mixer. After each addition of HGN, the suspension was left unstirred for 5 min. A total of 20 aliquots were added in this manner. Using this method, we estimate that on average one HGN is attached to each liposome. The encapsulation efficiency (∼5.3%) was calculated from the percentage volume within the liposomes compared with the total volume of drug solution used for suspending the lipids. To estimate release, we referred to previous work (Nakano et al., 2014) in which release from liposomes trapped on a carbon fiber was determined by measuring the oxidation current of dopamine. By fitting an exponential curve to a series of release events, the fraction of the cargo released at each event was estimated as 5.8%. The liposome suspension was then placed inside dialysis tubing and dialyzed with PBS buffer, which was stirred during dialysis.

### Laser stimulation

We used a two-photon microscope (FV1000, Olympus) to stimulate the liposomal nanostructures. The two-photon microscope has an infrared (890 nm) femtosecond laser, with pulse widths of 100 fs and a repetition rate of 80 MHz. Laser pulses were transmitted through a 60× or 40× objective lens and focused to a 430 nm spot size in the brain slice, which was then scanned over an area with dimensions of 212 × 212 µm (60×) or 318 × 318 µm (40×). Laser power at the source of 2 W is intensified at the sample (800 kW/cm^2^) and attenuated by an acousto-optic modulator. A single spot (pixel) had a dwell time of 2 µs (160 pulses). The laser stimulation setting was software controlled (FluoView, Olympus).

### Plasticity experiments

EPSPs were evoked in D1 receptors expressing medium spiny neurons in the striatum by extracellular stimulation with bipolar electrodes placed in the corpus callosum. Stimulation intensity (0.1–3.0 mA, 0.2 ms duration) was adjusted to evoke baseline EPSPs with amplitudes of 1–6 mV. Baseline EPSPs were recorded for 10 min at a 0.05 Hz stimulation rate.

After the baseline period, a plasticity-inducing stimulation was applied that consisted of 0.2 s suprathreshold postsynaptic current injections paired with 20 Hz electrical stimulation of the cortex to produce a presynaptic and postsynaptic conjunction of activity, which would normally produce long-term depression. Liposomal nanostructures were irradiated with a laser that commenced output from 0.4 s after the onset of the current injection and continued for 0.43 s. This conditioning stimulation protocol was repeated four times at 30 s intervals. The change in EPSP amplitude was evaluated by averaging the responses that were evoked in the period 10–20 min after the conditioning. For group averages, responses were expressed as the percentage change from the baseline EPSP amplitude. Data are presented as the mean ± SEM.

### Statistical analysis

Statistical analysis was undertaken to test the effectiveness of the liposomes, comparing baseline and evoked responses using Student’s *t* test, accepting a significance level of *p* < 0.0001. To determine the significance of the regression analysis, we tested the null hypothesis that the coefficient is equal to zero (no effect), using a significance level set to *p* < 0.001. For the plasticity experiments involving comparison across groups, analysis of variance (repeated measures design) using the general linear model procedures (SPSS, IBM) was used with α set to *p* < 0.05.

## Results

**Figure 1.**
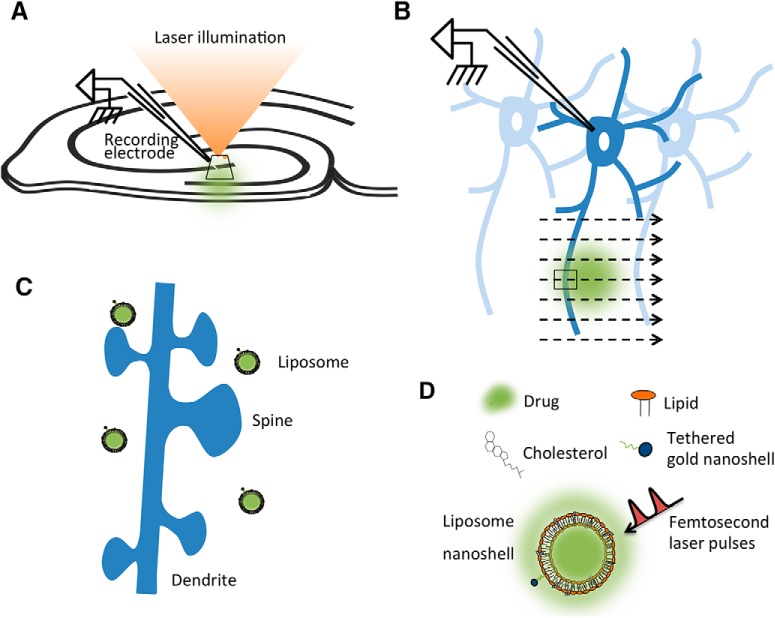
Experimental setup. ***A***, Diagram showing relative positions of recording electrode in hippocampus and laser illumination. Whole-cell recordings were made from pyramidal neurons using patch electrode positions in the liposome-injected region. ***B***, Enlargement of the area shown by the oblique rectangle in ***A***. Laser stimulation was applied to the pyramidal neuron dendritic region by scanning, so that the laser lit small regions one by one, with a laser dwell time of 2 µs. The small rectangle shows the laser scan crossing the dendrite in the region of liposome injection. ***C***, Enlargement of the small rectangle shown in ***B***, showing the location of liposomes in relation to dendrites and dendritic spines. Laser-stimulated liposomes release drugs close to dendritic spines. ***D***, Liposomes were assembled from components, as described previously. Adapted from the study by Nakano et al. (2014) with permission of the authors. Laser spot, Neuronal structures and liposomes not drawn to scale.

In order to test their effectiveness in live brain tissue, liposomal nanostructures were loaded with different agents and injected into area CA1 of the hippocampus. The volume injected was estimated to be 3–5 nl based on the diameter of the volume observed in bright-field images displayed on a monitor screen. Based on the liposomal formulation, we estimated 3.2 million liposomes per nanoliter. To measure release, we made whole-cell recordings from hippocampal CA1 pyramidal cells during exposure of the injected area to laser stimulation (Fig. 1*A*). The recorded cells were ∼500 µm distant from the center of the bolus injection, within the pore space of the injected material. As the laser scanned the area, the focal point of illumination passed over the injected liposomal nanostructures in the stratum radiatum (Fig. 1*B*). Laser-stimulated release of substances caused membrane potential responses, which differed according to the types of receptors or ion channels involved, and their relative location on dendritic spines and dendrites (Fig. 1*C*).

**Figure 2.**
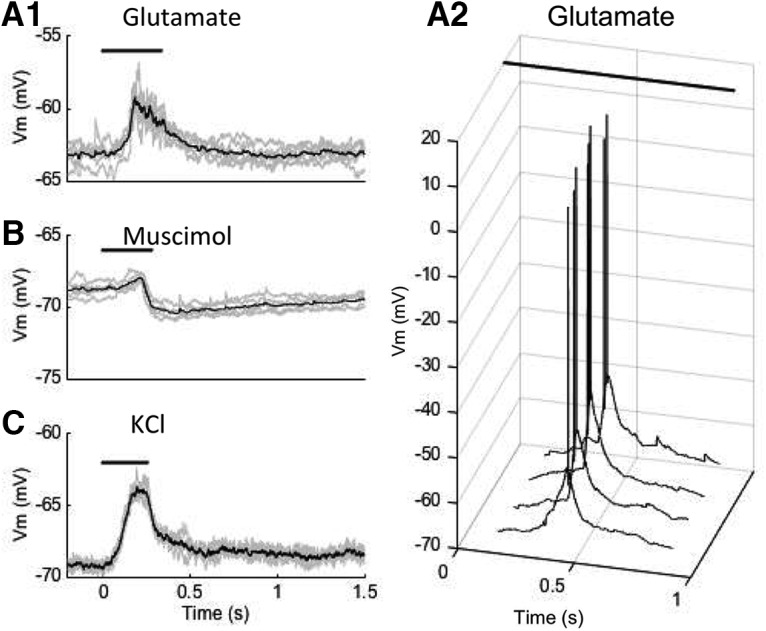
Whole-cell recordings of neural responses to laser stimulation of liposomal nanostructures. ***A1***, Subthreshold membrane potential depolarization in response to laser stimulation (320 kW/cm^2^) of glutamate-loaded liposomes. ***A2***, Action potential responses in response to stimulation at same intensity (320 kW/cm^2^), using a higher concentration of liposomes. ***B***, Hyperpolarizing responses to stimulation (800 kW/cm^2^) of liposomes loaded with the GABA agonist muscimol. ***C***, Depolarizing response to stimulation (8 kW/cm^2^) of KCl-containing liposomes. Each trace (***A1***, ***A2***, ***B***, and ***C***) shows the mean of four to six responses, with individual responses overlaid. Note that spot scanning may stimulate release at time points that are delayed relative to scan onset. Bar indicates the duration of a single scan.

When liposomes were loaded with the excitatory neurotransmitter glutamate, laser stimulation evoked a transient depolarization of the cell membrane, with a subsecond timecourse (Fig. 2*A1* ). Depolarizations (mean, 4.98 ± 0.41 mV; *p* < 0.0001 vs baseline; *n* = 6) could be evoked reliably and repeatably (>50 repetitions). Depolarizations occurred with constant latency from the onset of each scan period, consistent with release from one or more liposomes excited at a certain point in the scan. The same intensity of stimulation was able to evoke action potential firing of CA1 pyramidal cells when the injection volume was increased several fold (Fig. 2*A2*), presumably due to the release of greater quantities of glutamate.

To test the ability of the system to release a wider range of substances than previously possible, we tested liposomes loaded with the GABA_A_ receptor agonist muscimol, a synthetic compound not available for pulsatile application by electrical, optogenetic, or uncaging methods. Stimulation of muscimol-containing liposomes caused a transient hyperpolarization (mean, −1.87 ± 0.20 mV; *p* < 0.0001 vs baseline; *n* = 5) of the membrane potential (Fig. 2*B*) consistent with its known pharmacological actions mediated by binding to GABA_A_ receptors. This demonstrates the possibility of phasic release of a synthetic agent on a subsecond timescale.

To test their effectiveness in delivery of small ions, liposomes were loaded with potassium chloride. Lipid bilayer membranes are relatively impermeable to potassium chloride, which dissociates into K^+^ and Cl^−^ in water, both of which are charged and have relatively large hydration spheres. Published permeability coefficients for the passage of K^+^ and Cl^−^ through synthetic lipid bilayers are <10^−10^ cm/s (Alberts et al., 2002, their Fig. 11-2). Therefore, we tested whether laser stimulation of potassium chloride-containing liposome would cause release, detectable as changes in the membrane potential of the recorded cells. We found that laser stimulation caused neural depolarization (mean, 5.96 ± 0.28; *p* < 0.0001 vs baseline; *n* = 5) consistent with a change in the local extracellular concentration of potassium and a consequent shift in the equilibrium potential of the leak conductances of the cells (Fig. 2*C*). These responses were also repeatable >50 times. Together with the foregoing results, these findings suggest that the method has the potential to provide novel types of control over neural activity by pulsatile application of a broader range of neurochemicals, drugs, and ionic compounds than has previously been possible.

**Figure 3.**
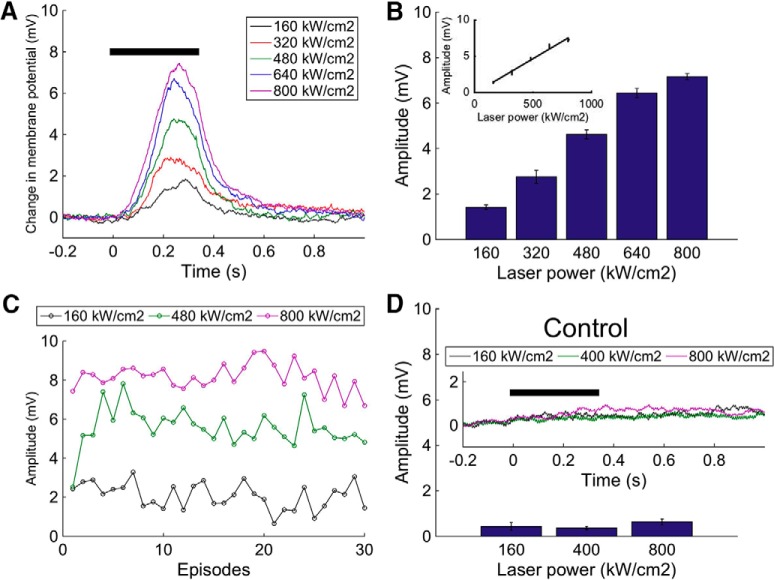
Repeated and amplitude-controlled release by liposome. ***A***, Subthreshold neural responses to different laser power. The black bar indicates the laser exposure. Each trace is the average of 30 responses. ***B***, Response amplitude for successively increased laser power, showing the average and SEM at each power level. There is a good fit to a regression line *y* = 0.0095x–0.075 with *R* ^2^ = 0.98. ***C***, Repeatability of subthreshold responses over 30 repetitions of the same stimulus at three different laser power settings. ***D***, Control experiment using PBS containing liposome shows small amplitude responses to laser exposure.

We then investigated the reliability of control of neural function by liposomal release using glutamate-containing liposomes (Fig. 3). We first measured the neural responses to the laser stimulation of glutamate-loaded liposomes as we varied the laser power, using a liposome concentration that produced subthreshold membrane potentials. We found that the amplitude of the responses changed systematically as we varied the laser power, with stronger laser stimulation causing a larger response amplitude (Fig. 3*A*), and, on average, were approximately linearly related (*r* ^2^ = 0.98, *p* < 0.001, *n* = 30) to the intensity up to 640 kW/cm^2^ (Fig. 3*B*). The subthreshold responses were repeatable over many trials (≥30) with stable amplitude (Fig. 3*C*), confirmed statistically by the lack of significant difference of the regression coefficient from zero at 160 kW/cm^2^ (*p* = 0.07, nonsignificant), 480 kW/cm^2^ (*p* = 0.47, nonsignificant), or 800 kW/cm^2^ (*p* = 0.41, nonsignificant).

At higher power settings, even though the exposure period is brief, it is possible that the intensity of stimulation may have direct effects on the membrane potential. In order to confirm that the responses were caused by the glutamate release from laser-stimulated liposome, and not by the direct effects of the laser on the neural processes or liposomes themselves, we exposed PBS-containing liposome to the laser as a control experiment. The laser stimulation to the PBS-containing liposome did not cause depolarizing responses (Fig. 3*D*), and there was no significant indication of an association of membrane potential with power level (*p* = 0.08, nonsignificant). Together with the significant effects of glutamate-loaded liposomes, these results show that liposomal drug application can be used repeatedly to cause membrane potential depolarizations of controlled amplitude, thus providing a novel means of interfacing light and neural activity that does not require genetic manipulation or depend on the chemical properties of the agents.

To investigate whether the liposomal nanostructures could be used to produce physiological changes by the release of specific dopamine agonist drugs, we tested the effectiveness of liposomal nanostructures in modulating activity-dependent synaptic plasticity in the striatum. Previously, the neuromodulator, dopamine—acting via dopamine D1 receptors—has been known to modulate synaptic plasticity in the striatum (Reynolds et al., 2001). However, tonic stimulation of dopamine D1 receptors by dopamine D1-selective agonist drugs does not have this effect. We hypothesize that the effect requires phasic activation of the receptor, because of the rapid desensitization of dopamine D1 receptors when constantly exposed to agonists (Ofori et al., 1993) and previous studies showing that pulsatile application, but not bath application, of dopamine causes long-term potentiation (Wickens et al., 1996).

To test this hypothesis, we used liposomes loaded with the dopamine D1 agonist SKF-38393, and laser stimulation to phasically activate the dopamine D1 receptors. Using corticostriatal slices from transgenic mice in which EGFP is selectively expressed in D1 receptor-expressing medium spiny neurons of the striatum, we measured EPSPs before and after an experimental treatment involving activity and phasic dopamine D1 receptor stimulation. EPSPs were evoked in the medium spiny neurons by stimulating the corpus callosum, and test responses were recorded before and after an experimental treatment.

**Figure 4.**
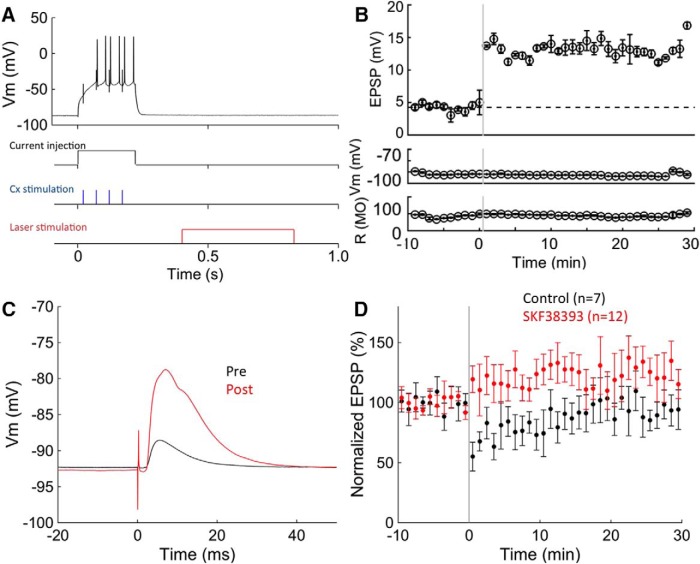
Dopamine D1 receptor-mediated synaptic plasticity. ***A***, The top trace shows membrane potential during conditioning. Lower traces show the conditioning stimulation protocol. Conditioning stimulation was a combination of cortical stimulation (four pulses at 20 Hz) and suprathreshold postsynaptic current injection paired with cortical stimulation. Laser stimulation (0.43 s scan, 2 µs dwell time at 400 kW/cm^2^) was delayed 0.4 s. ***B***, An example of the change in the synaptic responses. The synaptic response increased after the conditioning using SKF-38393 containing liposome, while resting membrane potentials and input resistance remain unchanged. ***C***, Examples of EPSPs before and after conditioning. The black line indicates preconditioning, and the red line indicates postconditioning corresponding to the bar in ***B***. ***D***, Group average of the change in the synaptic efficacy using SKF containing liposome (red) or ascorbic acid containing liposome (black). Values are reported as the mean ± SEM.

Liposomes loaded with SKF-38393 plus ascorbic acid in PBS (D1 agonist experimental group), or ascorbic acid alone in PBS (vehicle controls) were injected into the slice in advance of the recording. Ascorbic acid was used to reduce oxidation of the dopamine agonist. To induce synaptic plasticity, electrical stimulation of presynaptic inputs was repeatedly paired with firing of the postsynaptic neurons, a protocol that is expected to produce long-term depression based on previous studies (Wickens et al., 1996; Shindou et al., 2011). Phasic release of SKF-38393 plus ascorbic acid or ascorbic acid alone (Fig. 4*A*) was paired with the electrical stimulation. After pairing, the amplitude of EPSPs in the DA agonist group increased significantly relative to that of the controls, and remained increased for the duration of the recording (mean normalized EPSP 1–20 min after pairing: control, 0.81 ± 0.10 mV, *n* = 7; SKF-38393, 1.12 ± 0.08 mV, *N* = 11; *p* < 0.05, Fig. 4*B*,*C*), long outlasting the application of SKF-38393, indicating a modulatory effect on long-term plasticity of synaptic responses. The example cell shown exhibited a very large potentiation, possibly indicating a favorable location of liposomes relative to the synapses mediating the postsynaptic response, while other cells showed fewer dramatic changes, as indicated in the group averages (Fig. 4*D*, SKF-38393). The cell shown exhibited the largest potentiation seen, well above the average, was treated as an outlier, and was not included in the statistical analysis. In contrast, the amplitude of EPSPs decreased after conditioning using liposome loaded with only ascorbic acid (Fig. 4*D*, controls). These findings support the hypothesis that phasic activation of dopamine D1 receptors is sufficient for the induction of dopamine D1 receptor-dependent synaptic plasticity.

**Figure 5.**
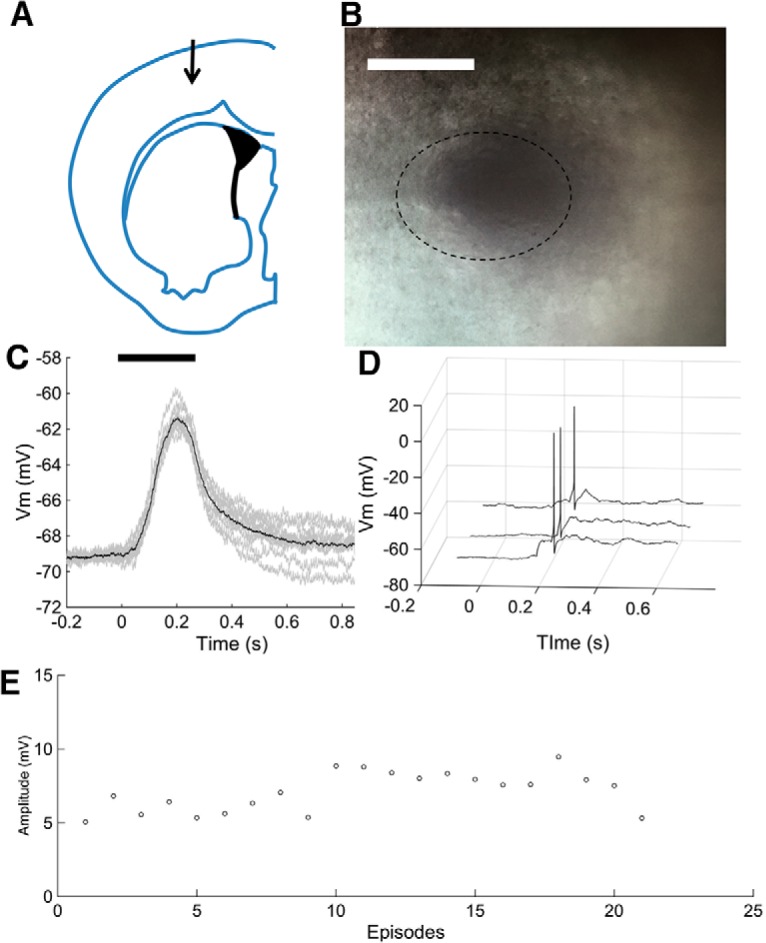
Liposome functionality after 1 week *in vivo*. ***A***, Liposomes were stereotaxically injected into cerebral cortex at the location shown. See text for stereotaxic coordinates. ***B***, Bright-field image of injected liposome in brain slice, after slice preparation and maintenance in the recording chamber. A dotted line encircles the area darkened by the presence of gold nanoparticles. Scale bar, 200 µm. ***C***, Subthreshold neural response repeatedly evoked by laser stimulation (8 kW/cm^2^). Dark trace shows the mean of 10 responses, with individual responses overlain. ***D***, Spikes repeatedly evoked by stimulating liposome by laser at higher power (160 kW/cm^2^) 1 week after stereotaxic injection into the mouse brain. ***E***, Repeatability of responses (0.254s, 2 µs dwell time, 8 kW/cm^2^) over 20 episodes (includes data from ***C***). Interstimulus interval, 30 s (***C–E***).

A crucial question is the suitability of the liposomes for use under *in vivo* conditions. To test the durability of liposome in live animals, we made injections of glutamate- and 6-CF-containing liposomes into the cerebral cortex stereotaxic coordinates [anteroposterior (AP), 1.1; mediolateral (ML), 1.5; dorsoventral (DV), 1.4; Fig. 5*A*], and tested functionality at 1 week and 1 month after the injection. One week after injection, following the preparation and superfusion of brain slices during maintenance and in the recording chamber, opacities indicating the presence of liposomes could be seen under infrared differential interference contrast illumination at low magnification (Fig. 5*B*). Whole-cell recordings from pyramidal neurons in these slices showed that laser stimulation at low power (8 kW/cm^2^) evoked subthreshold membrane potential depolarizations (mean, 7.12 ± 0.30 mV; *p* < 0.0001; *n* = 10; Fig. 5*C*). Suprathreshold responses were evoked at higher laser power (160 kW/cm^2^; Fig. 5*D*). As previously shown for acutely injected liposomes, the transient depolarizations could be evoked reliably and repeatedly over several trials (Fig. 5*E*). These findings show that liposomes survive in the brain of living animals and remain functional for at least 1 week.

**Figure 6.**
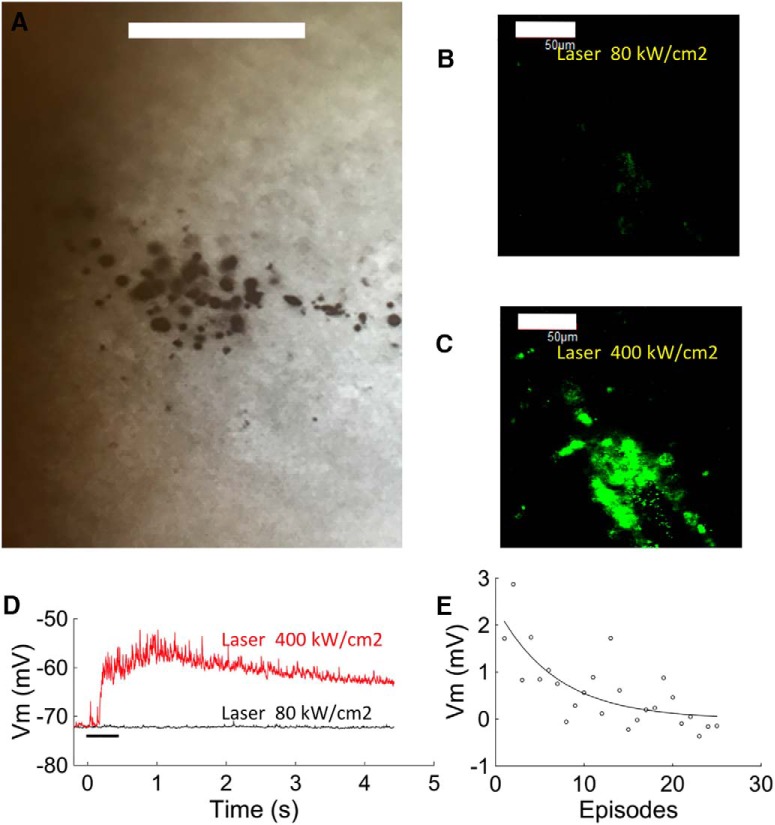
Survival of liposomes after 1 month *in vivo*. ***A***, Image of injected liposome in the brain slice. Dark material indicates the presence of gold nanoparticles. Scale bar, 200 µm. ***B***, ***C***, Two-photon images showing the fluorescence of released CF during laser scanning at 10% (***B***) and 50% (***C***) of maximum power. Scale bar, 50 µm. ***D***, Electrophysiological response of neurons recorded during laser stimulation in ***B*** (black trace) and ***C*** (red trace), showing depolarization in response to higher power. ***E***, Repeatability of response amplitude over 20 episodes (interstimulus interval, 30 s). The decrease in response amplitude fitted a single exponential decay (fitted curve, *y* = 2.41e−0.1488*x*).

One month after injection into a similar cortical region (AP, 0.6; ML, 1.5; DV, 1.5), opacities could still be seen (Fig. 6*A*). In slices made from these animals, laser stimulation at low power evoked minimal release of CF (Fig. 6*B*). Substantial release was observed at higher laser stimulation power (Fig. 6*C*), and this was associated with subthreshold depolarization (Fig. 6*D*). However, with repeated stimulation the response amplitude decreased over 25 release episodes, suggesting that after 1 month *in vivo* the proportion of fragile liposomes has increased (Fig. 6*E*).

## *D*iscussion

We have demonstrated that drug-encapsulated liposomes provide an interface with neural activity when stimulated by femtosecond laser pulses. This technique allows the encapsulation of a wide variety of neurochemically active substances, including glutamate, potassium chloride, and muscimol and specific dopamine agonists. By varying laser power, it is possible to control the release of neurochemicals, in turn modulating the neural responses. The evoked neural responses were repeatable and stable. Importantly, the liposomes are compatible with brain tissue and remain stable in the living brain for long periods.

The mechanism of light-induced release from gold-tethered liposome structures is not yet known, but there are several possibilities and clues from previous studies. We previously proposed that the following two different processes are involved: a process that may involve one-time destruction of a fragile subset of the liposomes; and a more common nondestructive and repeatable process in which the liposome wall becomes temporarily permeable to the contents and then reseals, allowing multiple release events. Paasonen et al. (2007) proposed that HGNs, via the linker molecules, may transfer heat to the liposomal wall and increase its fluidity. Similarly, Wu et al. (2008) suggest that release is caused by transient cavitation due to microbubble formation and collapse within the liposome wall. Both mechanisms involve steep temperature gradients that require femtosecond pulse irradiation. Importantly, the present formulation is dominated by the nondestructive process, as evidenced by SEM confirmation of the persistence of liposomal nanostructures before and after light-stimulated release (Nakano et al., 2014).

A particular advantage of this technique, compared with other optical stimulation techniques, is the ability to encapsulate a variety of chemicals. This allows the controlled release of not just intrinsic neurotransmitters, but also artificial molecules, such as selective receptor agonists, antagonists, and ion channel blockers. This enables the stimulation of particular subtypes of receptors or the blockade of specific ion channels, making a large arsenal of experimental tools available for reliable and repeatable delivery on a subsecond timescale. In comparison, optogenetic neural manipulation is limited to releasing naturally synthesized neurotransmitters from genetically modified neurons. While optogenetics provides unparalleled advantages for manipulating existing neural systems, it cannot substitute for neural functions that have been lost due to neurodegeneration or release novel, artificially synthesized pharmacological agents. Similarly, uncaging techniques are limited to compounds that have a molecular structure that is suitable for covalent binding to caging moieties, in turn limiting the possibilities of neural interfacing.

By varying laser intensity and pulse-timing parameters, laser-stimulated release from liposomes can be flexibly controlled. This flexibility is an advantage over caged compounds, which have a threshold-like, nonlinear characteristic due to the photolytic processes involved in uncaging, and do not produce smoothly graded variation in release amount. The graded release that is possible from liposomes thus provides a wider and more continuous range over which neuronal function can be manipulated.

The combination of spatiotemporally controlled release, along with the variety of available neurochemicals, also allows for other novel studies of neural functioning, which are not possible with existing methods. Further developments in the architecture of the gold nanoparticles tethered to the liposomes may enable selectivity in excitation wavelengths, such that, in principle, one could inject two populations of liposomes, both loaded with different neurochemicals, that respond to different wavelengths of light. This allows the possibility of multichannel stimulation. Last, we note that liposomes are highly biocompatible, and are not toxic to living tissue. We have demonstrated that the liposomes survived and stayed at the injected site for weeks while retaining the ability to release the encapsulated neurochemicals. These characteristics are well suited for *in vivo* experiments and provide the possibility of addressing neurological diseases in the future.

## Synthesis

Reviewing Editor: Upinder Bhalla, National Center for Biological Sciences The decision was a result of the Reviewing Editor Upinder Bhalla and the peer reviewers coming together and discussing their recommendations until a consensus was reached. A fact-based synthesis statement explaining their decision and outlining what is needed to prepare a revision is listed below. The following reviewers agreed to reveal their identity: Balaji Jayaprakash, Praveen Vemula

Synthesis:

The reviewers both felt that the reported method for localized release of

molecules would be useful. They did bring up a number of concerns about the

reporting and characterization of the method.

1. There is apparent copying of some of the methods section from a previous

publication. Even though the earlier work was cited, this is a serious

concern and all the authors must take responsibility to avoid this situation.

All wording and figures must be original, A less desirable possibility is to

explicitly quote an excerpt and state that the given section (or figure) is

reproduced from cited previous work. To do this one would need copyright

permissions.

2. The methods are insufficiently described. The authors must provide complete

information for reproduction of the experiments.

3. Images of the site of injection of the liposomes must be provided and

further details of the liposome release should be added.

4. More details are needed for the report of liposomal release mechanism. This

may require further (minor) experiments to quantify this.

5. There are several issues with respect to the formatting and labeling of

figures.

-----------------------------------------------------

Reviewer 1

This manuscript describes and utilizes a new method for localized release of drugs and other small molecules in neurons. The method by itself may be very useful and the authors do indeed show a few applications. However, the description of the method and description of how the whole lipposomal nano structures are prepared is lacking. Further there were a few places where the claims have been made (vital for the proof of principle) based on data that is not shown in the paper. Over all the method has good potential since the existing methods are limited in the range of molecules that can be released. The authors have demonstrated the usefulness and versatility of the method through releasing a variety small molecules. Eventhough many of the effects resulting from the experiments are large the authors have not described/done any statistical analysis to show that it is really significant. Also, it is important to rewrite the manuscript to address the folllowing minor comments:

Page 5-6

Methods section:

Preparation of liposomal nanostructures ;

Lacks adequate description of how the HGN is added and in what ratios. The wording of this section is too close to that provided in Nakano et al 2014 and should be changed. It is ncessary to provide complete and a clear description of what is being prepared and how it is done. For e.g. what buffer solution is used for encapsulation of Glutamate, Muscimol, Dopamine agonist.

Page 10:

"Responses could also be evoked from slices prepared one week after injection of liposomes but their amplitude was much reduced."

Do they mean one month ?

Is there any data for showing functionality after one month? It is not sufficient to say data not shown. At least for this claim the authors need to prove it convincingly.

Page 11:

Using liposomal neural manipulation, rapid, repeated and on-demand stimulation enables arbitrary manipulation of neural function, allowing quantitative experiments.

I am not sure what the authors mean by arbitrary ? Depending on the claim again we need new /may be already described in the manuscript.

Figure 1 : The 1A, 1B, 1C subsections are not marked. Parts of the figure are adapted from Nakano et al, 2014. Please mention the figure credits.

Figure 2. 2A2 Does not show action potential stimulation due to stronger laser stimulation/power as mentioned in the Figure legend (The power is 0.8 Watts in both 2A1 and 2A2 ) but it does so due to 'higher' concentration of liposomes (How high is high ?)

Figure 3B Graph does not show the linear fit as mentioned in the manuscript.

Figure 4 Given that the control ie. liposomes loaded with ascorbic acid showed a decrease in EPSP, a control with PBS loaded Liposomes, as has been shown in previous experiments would be useful.

Figure5. Where in the cortex were the liposomes injected stereotaxically? In section 5a, which region is this picture from? what is the scale? It might be preferable to show image of the brain region with neurons clearly stained with an inset showing gold nano particles (even as a diffraction limitedspots).

Since the liposomes should be extracellularly present, are they not washed off during slice preparation and maintenance ?

Other concerns:

There are some parts of the manuscript (as indicated in my review) where either the text or the figures are reproduced from other published manuscripts. For e.g. some of the statements in method section are an exact match to other (referenced) work.

------------------------------------------------------------

Reviewer 2.

Advances the Field. In this manuscript, authors have demonstrated that laser stimulation induced release of neurochemicals through liposomes can control the neural activity in a spatiotemporal manner. This is an interesting approach to control the neural activity. Although it still demands the surgical injection of liposomes, it is an interesting approach to control the neural activity in an on-demand manner.

Visual Abstract Visual abstract is informative, but it can be improved. For example, what changes occur on liposomes to release the drug upon exposed to femtosecond laser should be described.

In this manuscript, authors have demonstrated that laser stimulation induced delivery of neurochemicals through liposomes can control the neural activity in a spatiotemporal manner. This is an interesting approach to control the neural activity. Although conceptual demonstration is satisfactory, there are few major concerns authors need to clarify before considering for publication. This system needs to more robust characterization.

- The release mechanism needs to be described in detail. What are the changes are occurring while gold nanoparticle tethered liposomes exposed to femtosecond laser pulses? Is liposomal membrane getting disrupted (melted), which is causing the release of neurochemicals from the core of liposome?

- This entire release phenomenon should be demonstrated in a cuvette. For example, what is encapsulation efficiency of these liposomal? In response to a given pulse, how much cargo is getting released?

- The non-specific release of cargo under PBS conditions should be quantified. It is hard to imagine that salts like KCl are not diffusing through these liposomal membranes, thus showing their release in vitro will be critical.

- Are these liposomes unilamellar or multilamellar? Transmission electron microscope images should be provided.

- It is interesting to see that post intra-brain injection of liposomes; they retained in the brain up to one month without dissociating or releasing the cargo. Authours mentioned that presence of the liposomes was characterized by confocal imaging, but data is not given. I think it critical to include those images.

-----------------------------------------------------------------------------
